# Inefficient white matter activity in Schizophrenia evoked during intra and inter-hemispheric communication

**DOI:** 10.1038/s41398-022-02200-9

**Published:** 2022-10-16

**Authors:** Niccolò Zovetti, Marcella Bellani, Asadur Chowdury, Franco Alessandrini, Giada Zoccatelli, Cinzia Perlini, Giuseppe K. Ricciardi, Carlo A. Marzi, Vaibhav A. Diwadkar, Paolo Brambilla

**Affiliations:** 1 Department of Neurosciences, Biomedicine and Movement Sciences, Section of Psychiatry, University of Verona, Verona, Italy; 2 Department of Psychiatry & Behavioral Neurosciences, Wayne State University, Detroit, MI USA; 3 Neuroradiology Department, Azienda Ospedaliera Universitaria Integrata di Verona, Verona, Italy; 4 Department of Neurosciences, Biomedicine and Movement Sciences, Section of Clinical Psychology, University of Verona, Verona, Italy; 5 Pathology and Diagnostics, Section of Neuroradiology, Hospital Trust Verona, Verona, Italy; 6 Physiology and Psychology Section, Department of Neuroscience, Biomedicine and Movement Sciences, University of Verona, Verona, Italy; 7 National Institute of Neuroscience, Verona, Italy; 8 Department of Neurosciences and Mental Health, Fondazione IRCCS Ca’ Granda Ospedale Maggiore Policlinico, Milan, Italy; 9 Department of Pathophysiology and Transplantation, University of Milan, Milan, Italy

**Keywords:** Schizophrenia, Diagnostic markers

## Abstract

Intensive cognitive tasks induce inefficient regional and network responses in schizophrenia (SCZ). fMRI-based studies have naturally focused on gray matter, but appropriately titrated visuo-motor integration tasks reliably activate inter- and intra-hemispheric *white matter pathways*. Such tasks can assess network inefficiency *without* demanding intensive cognitive effort. Here, we provide the first application of this framework to the study of white matter *functional* responses in SCZ. Event-related fMRI data were acquired from 28 patients (nine females, mean age 43.3, ±11.7) and 28 age- and gender-comparable controls (nine females, mean age 42.1 ± 10.1), using the Poffenberger paradigm, a rapid visual detection task used to induce intra- (ipsi-lateral visual and motor cortex) or inter-hemispheric (contra-lateral visual and motor cortex) transfer. fMRI data were pre- and post-processed to reliably isolate activations in white matter, using probabilistic tractography-based white matter tracts. For intra- *and* inter-hemispheric transfer conditions, SCZ evinced *hyper*-activations in longitudinal and transverse white matter tracts, with hyper-activation in sub-regions of the *corpus callosum* primarily observed during inter-hemispheric transfer. Evidence for the functional inefficiency of white matter was observed in conjunction with small (~50 ms) but significant increases in response times. Functional inefficiencies in SCZ are (1) observable in white matter, with the degree of inefficiency contextually related to task-conditions, and (2) are evoked by simple detection tasks without intense cognitive processing. These cumulative results while expanding our understanding of this dys-connection syndrome, also extend the search of biomarkers beyond the traditional realm of fMRI studies of gray matter.

## Introduction

The clinical presentation of schizophrenia (SCZ) has been much discussed and extensively characterized for over 100 years. In terms of its etiology, multiple genetic, environmental, and developmental factors appear to play a role in the condition’s emergence [1], though no single factor appears necessary or sufficient. Expectedly, the expansion of in vivo methods for structural and then functional imaging has motivated a focus on its neurobiological bases. This focus has been on discovering alterations in brain structure and structural brain networks [2–8], but more recent work has advocated for focus on dys*functional* brain networks, primarily using fMRI data [9–12].

The heavy use of fMRI is natural given the method’s favorable balance (relative to its natural competitors) of spatial and temporal resolution [13]. This balance supports inferences regarding both spatial localization (e.g., *“where are differences in brain activity observed in SCZ”*) and network effects (e.g., *“between which regions do we see differences in connectivity in SCZ*”), largely based on time series analyses [14]. Early positron emission tomography (PET) studies on activation-based differences in SCZ were largely motivated by the notion of “hypo-frontality”, the reasonable idea that the illness was underpinned by a loss of prefrontal function [15, 16]. Subsequent fMRI studies using prefrontal-targeted tasks (e.g., working memory) appear to have replicated some of these effects but were beset with interpretational challenges [17]. First, generalized performance deficits in patients impair both task engagement and behavioral performance on effortful tasks like working memory. Such intergroup differences in task engagement can in turn occlude the meaningful interpretation of activation-based differences [18]. Because fMRI signals are strongly modulated by task engagement [19], reductions in fMRI-measured activation in SCZ were likely to reflect a loss of engagement, rather than a *true* loss of neuronal function. Indeed, subsequent investigations have affirmed this effect: generally, SCZ patients show *greater* fMRI-measured activation when task-performance or engagement is normalized [17, 20]. These findings have suggested that the SCZ brain is characterized by inefficient cortical functional processing [21–24]. Cortical inefficiency is thought to reflect compensatory generalized neural effects that presumably offset deficits evoked by the illnesses clinical symptomatology [25].

The cortical inefficiency hypothesis presents at least two corollary questions that to our knowledge, have not been addressed: (1) Does inefficiency present itself even during basic sensori-motor processing, and (2) can signatures of inefficiency be detected in the functional pathways that connect cortical regions, i.e., white matter tracts? If the answer to the first question is in the affirmative, it opens new vistas for studying disordered functional neurobiology in SCZ. And, if the answer to the second question is in the affirmative, then it suggests that white matter dys-*function* should be a viable bio-marker of the illness. Such evidence will augment the substantial literature showing altered structural integrity of white matter in SCZ [26, 27], and can indicate that the sources of this “dys-connection syndrome” are highly multifarious in origin [10].

The current study addressed these corollary questions by analyzing fMRI data collected in a well-characterized sample of SCZ patients (and roughly matched HC), while participants engaged in the Poffenberger paradigm [28]. This historically significant visuo-motor integration task was originally designed to psychometrically investigate the reaction time costs of inter-hemispheric over intra-hemispheric transfer [29]. This is achieved through the tachistoscopic presentation of visual probes to the left or right visual hemi-field (thus, stimulating the contra-lateral visual cortex) while requiring participants to signal the detection of the probe with the left or right hand (thus, relying on the motor cortex that is ipsi- or contra-lateral to the stimulated visual cortex). The task is rapid inter-hemispheric transfer is independent of effects such as memory load [30] and is therefore ideal for our goals. Moreover, multiple independent studies have affirmed that the paradigm reliably evokes fMRI-measured activation in longitudinal and transverse white matter tracts, including engagement of regions in the corpus callosum [31–33]. That the paradigm reliably targets the corpus callosum is particularly important, given that the structure is the principle inter-hemispheric commissure [34, 35], and that its macro-structure and integrity are impaired in SCZ [36–38].

## Materials and methods

### Participants

Twenty-eight SCZ patients (nine females, mean age 43.25, ±11.66) and 28 healthy controls (nine females, mean age 42.04 ± 10.14) provided informed consent to participate. All study procedures were approved by the institutional review board at the University of Verona, Italy. Table 1 provides demographic information for the sample.

**Table 1:** Demographic information and clinical characterization of patients and healthy controls.

Variables	SCZ	HC	Statistic	*p* value
Sex (F/M)	9/19	9/19	*χ*^2^ = 0	1
Age, mean (sd)	43.25 (11.66)	42.04 (10.14)	t = 0.40	0.68
Education, years, mean (sd)	8.82 (2.49)	13.85 (4.84)	t = −4.61	<0.01
Length of illness, years, mean (sd)	14.75 (11.18)	---	---	---
GAF, mean (sd)	52.68 (11.42)	79.08 (6.45)	t = −10.34	<0.01
BPRS, positive symptoms, mean (sd)	9.32 (2.98)	---	---	---
BPRS, negative symptoms, mean (sd)	8.22 (4.6)	---	---	---

*BPRS* Brief Psychiatric Rating Scale, *GAF* global assessment of functioning scale; *HC* healthy controls; *SCZ* Schizophrenic patients; *sd* standard deviation.

SCZ patients were recruited at the psychiatric centers of Verona. Diagnosis of SCZ was confirmed prior to enrollment into the study through the Structured Clinical Interview for DSM-IV conducted by trained psychiatrists [39]. Participants with cognitive disabilities, substance or alcohol use disorders, neurological illness were excluded. Positive and negative symptoms were assessed with the Brief Psychiatric Rating Scale (BPRS) scale. Global assessment of functioning (GAF) was assessed through the GAF scale. Average BPRS scores (positive and negative symptoms) were 9.32 (±2.98) and 8.22 (±4.6), respectively. The average patients’ GAF score was 52.68 (±11.42). All participating patients had been stabilized on a regime of antipsychotic medication at the time of the scan. Table 2 shows medication dosage at the time of the scan expressed as a prescribed daily dose (PDD) and defined daily dose (DDD) ratio (PDD/DDD) [40].

**Table 2:** Patient medications and dosage.

Atypical antipsychotics	Number of patients	PDD/DDD, mean (sd)
Clozapine	4/28	1.02 (0.51)
Risperidone	2/28	1.33 (0.93)
Quetiapine	3/28	0.75 (0.35)
Olanzapine	9/28	1.44 (0.72)
Chlorpromazine	1/28	13.33
Typical antipsychotics		
Haloperidol	8/28	0.65 (0.95)
Fluphenazine	1/28	1.66
Antidepressants		
Citalopram	5/28	1.21 (0.6)
Escitalopram	1/28	1
Paroxetine	2/28	0.7 (0.3)
Sertraline	2/28	0.75 (0.25)

*DDD* defined daily dose; *PDD* prescribed daily dose, *sd* standard deviation.

Note: medication dosage is expressed as PDD/DDD ratio.

### fMRI data acquisition

MRI data were acquired on a 3-Tesla Siemens Allegra system (Siemens, Erlangen, Germany) with a standard head coil. T2*-weighted images were acquired using a gradient-echo EPI-BOLD pulse sequence (TR: 2000 ms; TE: 30 ms; flip angle 75°; FOV: 92 × 192; 31 axial slices; thickness: 3 mm; in-plane: 3 mm^2^; matrix: 64 × 64). High-resolution MPRAGE T1-weighted structural images were acquired in the same session (TR: 2300 ms; TE: 3.93 ms; flip angle 12°; FOV: 256 × 256; 160 axial slices; slice thickness: 1 mm; matrix 256 × 256).

### Poffenberger paradigm

**Fig. 1:**
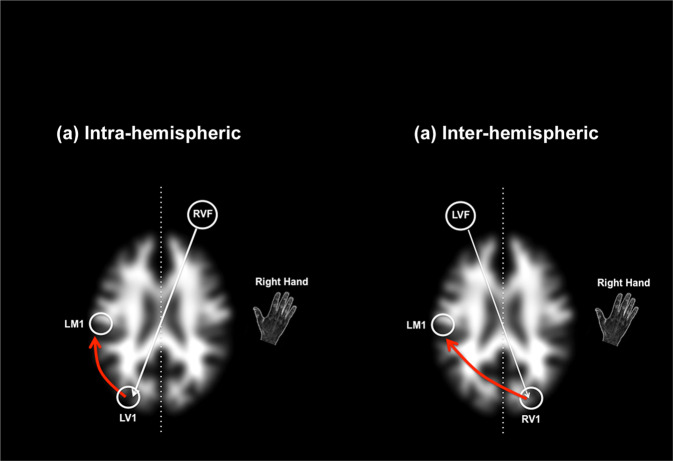
Schematic task depiction. The Poffenberger Paradigm depends on the brief stimulation of one or the other visual hemi-field by a briefly presented probe, detection of which must be signaled by the right hand. The arrows schematically depict task flow under **a** within-hemispheric conditions, when the probe stimulates the visual cortex that is within the same hemisphere as the responding motor cortex (note that the red arrow does not cross the mid-line), and **b** inter-hemispheric conditions, the probe stimulates the visual cortex that is in the opposite hemisphere as the responding motor cortex (note that the red arrow crosses the mid-line). In the current study response hand was maintained to the dominant right hand, therefore within and inter-hemispheric conditions were created by presenting the probe in the right or left visual field.

During fMRI, participants were positioned with adjustable padded restraints employed for head stabilization. Subjects maintained fixation on a centrally positioned marker, while probes were briefly presented (100 ms, pre-empting saccadic eye movements) for detection. Probes were ~1° in size appearing at a retinal eccentricity of ~7° along the horizontal meridian of one or the other visual hemi-field. Stimuli were rear-projected using an IFIS-SA presentation system (MRI Devices), and subjects were instructed to respond as quickly as possible to signal probe detection. Responses were collected using a button response unit. Design efficiency for fMRI was maximized using event-related probe presentation [41–43], with jittering of successive intervals (3–6 s randomly applied). Thirty event trials were collected per condition. Figure 1 provides a lucid conceptual representation of the task along with visual depictions of the bases of the intra- and inter-hemispheric conditions. The underlying brain image is a transverse section of a segmented and smoothed white matter template.

### fMRI processing

fMRI and T1 data were processed in SPM12 using established methods for temporal (slice timing correction) followed by spatial preprocessing. First, the structural and functional images were manually reoriented to the AC-PC axis. The structural images were segmented to identify different tissue classes using SPM12’s tissue probability maps, creating a nonlinear deformation normalization model. Motion correction and alignment to the template image were followed by co-registering the EPI images to the anatomical. The deformation model previously created was applied to the co-registered images to normalize them to the MNI space [44]. A low pass filter (128 s) was used to remove noise related to low-frequency components and the warped images were smoothed using a *relatively narrow* (4 mm full width at half maximum) Gaussian filter. Smoothing transforms the data to fulfill the statistical requirements of parametric statistics within the context of random field approaches, while the narrow filter maintained relative localization of signal, thus allowing us to better fulfill our aims of exploring activations of white matter [45].

First-level GLM analysis employed regressors to represent conditions (Inter- or Intra-hemispheric transfer), modeled as individual events convolved with a canonical hemodynamic reference waveform, with time and dispersion derivatives (to allow for peak, and subject- and voxel-wise variations, respectively). The six motion parameters (three for translation and three for rotation) were included in first-level models as “regressors of no interest” to filter out motion-related contributions to the signal. Each participant contributed two contrasts to a second-level random effects analyses of co-variance using Group (HC vs SCZ) as an independent factor and Condition (Inter vs. Intra) as a non-independent factor, with participant age modeled as a co-variate. In the factorial design, directional contrasts were employed to quantify inter-group differences in activation associated with each condition. This analytical approach allowed us to directly compare the effects of uni-manual responses in each of the inter- and intra-hemispheric transfer conditions.

Significant clusters within each region identified using AlphaSim [46], were derived by estimating the minimum cluster extent for activated clusters to be rejected as false positive (noise-only) clusters (family-wise error corrected using a frequency of occurrence of minimum cluster thresholds of *α* < 0.05  from 10^4^ Monte Carlo simulations). This approach performs a Monte Carlo alpha probability simulation, computing the probability of a random field of noise (after accounting for the spatial correlations of voxels based on the image smooth-ness within each region of interest estimated directly from the data set) to produce a cluster of a given size, after thresholding the noise at a given level. Thus, instead of using only the individual voxel probability threshold in achieving the desired overall significance level, the method uses a combination of both probability thresholding and minimum cluster size thresholding. The underlying principle is that *true* activation will tend to occur over contiguous voxels within a region of relative functional homogeneity, whereas noise has much less of a tendency to form clusters. This method has reliably identified activation clusters within narrowly defined motor regions [47, 48] during simple motor behavior.

### Reliably isolating activations in white matter

Activations in white matter were isolated using Johns Hopkins University’s (JHU) whole brain white matter probabilistic map [49, 50]. This mask was originally created based on Diffusion Tensor imaging data from control participants and provides a stereotactic representation of reliably classifiable white matter tracts. For 3D tract reconstruction, the Fiber Assignment by Continuous Tractography (FACT) method was used with a fractional anisotropy threshold (0.2) in DTIstudio. Tracts are reconstructed using multiple regions of interest and leveraging existing anatomical knowledge, with two different templates (JHU-DTI and MNI-ICBM152) used for spatial normalization and co-registration. These maps of eleven white matter tracts reveal high probabilities within a skeletal core, while probabilities are more dispersed and less reliable in the vicinity of the cortex itself (and are therefore excluded from the mask). Twenty distinct white matter tracts were identified in MNI space using this DTI approach, and for the present study, the map was binarized, to restrict the identification of white matter-related activations to regions more reliably classified within this tissue class.

### Analyses of response latencies

Mean response latencies for all participants were analyzed in a two-way mixed analysis of co-variance (SPSS 20) with group as a between-subjects factor and condition as a within subject’s factor (Age and gender were modeled as covariates). Data were visually and statistically inspected to check for normality. Sphericity was inspected with Mauchly’s test, and Greenhouse–Geisser corrections were used. The presence of outliers in the RTs was assessed through visual inspection (box plots), that is by calculating Tukey’s Fences; values above or below the fences were considered outliers and excluded from further analyses.

## Results

### Response latencies

Data from two HC participants were characterized by extremely low RTs across both conditions (two standard deviations below the mean) and were excluded from the analyses as outliers. The average rates of missing responses were 3% and 1% for the SCZ and HC groups, respectively (Missing responses were not replaced).

**Fig. 2:**
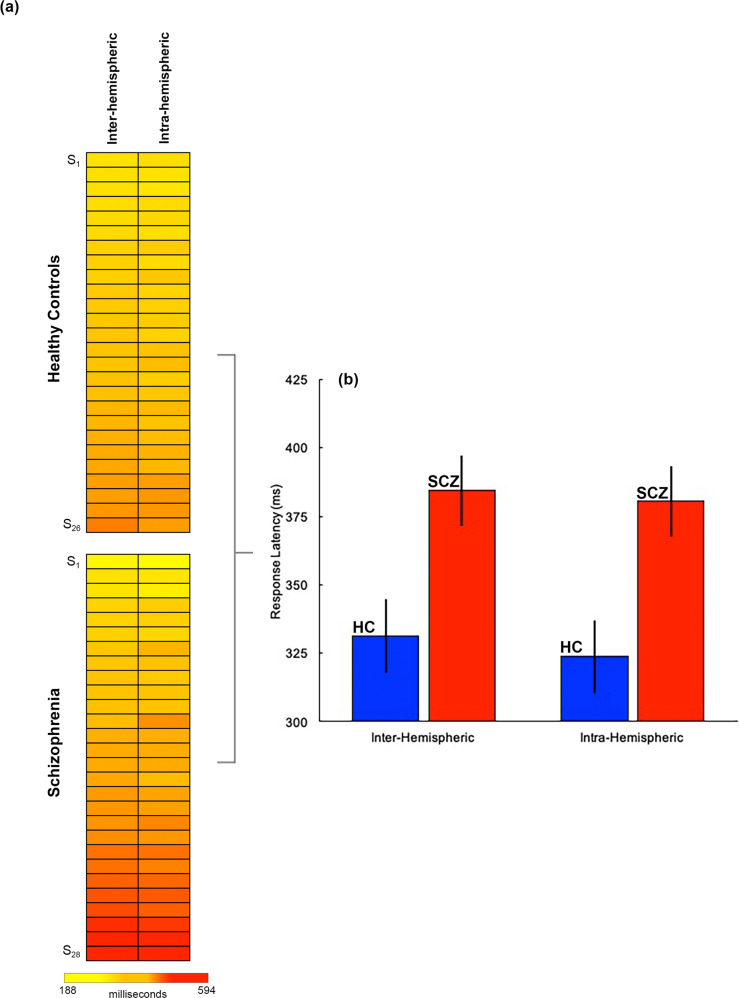
Behavioral results. **a** Response latencies across all healthy controls (*n* = 26) and patients (*n* = 28) are depicted in heat maps. In each group, data are arranged from fastest (top) to slowest (bottom) responses. **b** The data from a summarized in bar graphs (error bars ± s.d.) suggest a performance “cost” of ~50 ms in patients across both conditions (see Results for statistical information). As will be seen in Figs. 3 and 4, this relatively subtle cost in performance efficiency evinces highly exaggerated impacts on the activation-based differences.

The heat maps in Fig. 2a depict mean latency data for all participants included in the analyses of response latency data, and for both conditions. The bar graphs (b) depict mean latencies by condition and group (±s.d.).

The analyses of co-variance revealed a highly significant effect of group (*F*_*1,50*_ = 44.23; *p* < 0.001] with a large effect size (partial *η*^2^ = 0.47) and high power (1−*β* > 0.99) given the sample size of the analysis. The effect of the condition was not significant (*F*_*1,50*_ = 0.54; *p* > 0.10), revealing a small effect size (Partial *η*^2^ = 0.011) or a significant interaction (*F*_*1,50*_ = 0.62; *p* > 0.10).

In post-hoc analyses, multivariate linear regression (using age as a co-variate of no interest) was employed to evaluate if response latencies were affected by clinical variables including BPRS-negative and positive scores, GAF and length of illness. No significant associations were observed (Table 3).

**Table 3:** The table presents the results from applying multiple linear regression models to investigate the effects of clinical variables on response latencies in patients.

	B coefficient	*t*	Standard error	*p* value
Inter-Hemispheric latencies				
BPRS-positive symptoms	−2.95	−0.62	4.73	0.53
BPRS-negative symptoms	−2.03	−0.29	7.03	0.77
GAF	2.30	1.40	1.63	0.17
Illness duration	2.62	1.55	1.69	0.13
Age	3.13	1.88	1.66	0.07
Intra-hemispheric latencies				
BPRS-positive symptoms	−3.42	−0.72	4.73	0.47
BPRS-negative symptoms	−2.14	−0.30	7.03	0.76
GAF	1.39	0.85	1.63	0.40
Illness duration	2.87	1.70	1.69	0.10
Age	2.66	1.60	1.66	0.12

Results are separately documented for each of the Inter- and Intra-hemispheric conditions. The clinical variables included in each model were illness duration, GAF total score, BPRS-negative total score, BPRS-positive total score.

### fMRI results

**Fig. 3:**
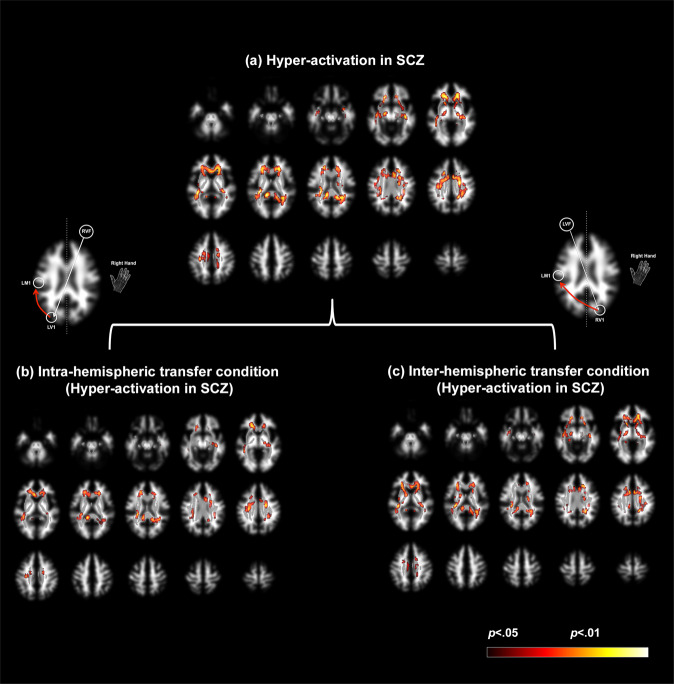
Hyper-activation of white matter pathways in schizophrenia. Hyper-activated clusters are depicted on a mosaic of axial views on a segmented white matter template in stereotactic space. The binarized tractography mask is visible as an overlay on the template (in grayscale). Inspection of the mask emphasizes its circumscribed nature relative to all of the segmented white matter. Clusters are overlaid on the tractography mask. **a** Under the main effect group, schizophrenia patients were characterized by significant patterns of hyper-activation in longitudinal and transverse white matter tracts. These patterns were unpacked within each of the **b** within-hemispheric and **c** inter-hemispheric conditions. The degree of hyper-activation scales by the task condition, with greater hyper-activation, observed under the inter-hemispheric condition. The patterns of hyper-activation in mid-line callosal structures are further detailed in Fig. 4.

Figure 3 depicts the comprehensive assessment of the 2nd level random effects analyses. In all the panels, significant clusters are overlaid on a mosaic of sixteen contiguous transverse slices (top left: inferior, bottom right: superior) of the segmented and smoothed white matter template. For clarity, the tractography-based probability white matter mask derived for the analyses is overlaid on the template in light grayscale, and is clearly seen as a spatial subset of the template. Finally, significant clusters (see Methods) are overlaid.

The results are organized in Fig. 3 as follows: (a) we first depict patterns of hyper-activation under the overall main effect of the group. Next, each of the condition-related effects under the overall main effect is depicted for (b) the Intra-hemispheric and (c) the Inter-hemispheric conditions. Statistically, relevant information is provided in Table 4, with effect size estimates (Cohen’s *d*) from the observed peaks ranging from 0.97 to 1.39, suggesting observed power (1−*β*) with the total sample size of 56 to be in ≥0.95 [51].

**Table 4:** Statistical information associated with the results presented in Figs. 3 and 4.

	Main effect (SCZ vs HC)					Intra-hemispheric (SCZ vs HC)					Inter-hemispheric (SCZ vs HC)			
	Right hand					Right hand					Right hand			
	Extent	*p*_unc_	*t*	Peak voxel		Extent	*p*_unc_	*t*	Peak voxel		Extent	*p*_unc_	*t*	Peak voxel
Retrolenticular part of Internal Capsule	2880	<0.001	4.95	[−30 −30 16]	Forceps Major	217	<0.001	3.87	[−14 −42 12]	Posterior Corona Radiata	965	<0.001	4.59	[22 −48 30]
Cingulum	560	<0.001	4.62	[8 8 30]	Superior Longitudinal Fasciculus	761	<0.001	3.68	[−28 −16 40]	Cingulum	337	<0.001	4.34	[−6 12 30]
Forceps Major	296	<0.001	4.04	[−14 −42 12]	Cingulum	106	<0.001	3.57	[8 10 28]	Retrolenticular part of Internal Capsule	2697	<0.001	4.28	[−38 −32 4]
Posterior Corona Radiata	868	<0.001	4.02	[22 −48 30]	Anterior Corona Radiata	783	<0.001	3.46	[−18 30 −6]	Retrolenticular part of Internal Capsule	288	<0.001	3.47	[34 −30 4]
Retrolenticular part of Internal Capsule	313	<0.001	3.45	[34 −30 4]	Superior Corona Radiata	361	<0.001	3.44	[22 −4 30]	Cingulum	77	0.001	3.23	[−8 −14 38]
					Superior Longitudinal Fasciculus	495	0.001	3.16	[38 −34 26]	Splenium of Corpus Callosum	277	0.002	3.02	[−22 −52 22]
					External Capsule	455	0.002	3.02	[16 30 2]	Uncinate Fasciculus	76	0.002	2.92	[36 −2 −18]
					Sagittal Stratum	197	0.002	2.95	[40 −24 −12]					

**Fig. 4:**
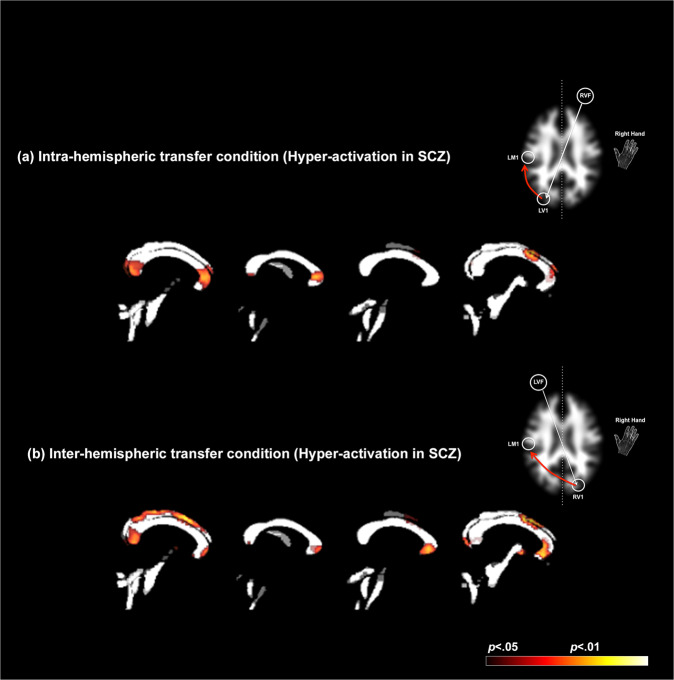
Hyper-activation during within and inter-hemispheric transfer in schizophrenia. Patterns of hyper-activation are rendered on four consecutive sagittal mid-line slices of the binarized white matter mask used to localize activations within longitudinal and transverse white matter tracts (see Methods). Two effects are evident: First, for both the **a** intra-hemispheric and **b** inter-hemispheric conditions (depicted by schematic figures on the right), schizophrenia patients are characterized by hyper-activation in mid-line callosal structures, including the genu, body, and splenium. Secondly, the extent of hyper-activation is amplified in the inter-hemispheric condition, suggesting that the network demands of this condition may be more sensitive in modulating hyper-engagement in schizophrenia of callosal pathways, including the splenium, frontal, motor, and visual areas across the hemispheres.

Multiple effects are observed. First, *regardless of condition*, patients are characterized by patterns of hyper-activation with peaks labeled in multiple locations including the retrolenticular portion of the internal capsule, the cingulum bundle, the forceps major, and the posterior corona radiata (labels are omitted from figures to reduce clutter). Under condition-specific contrasts (Intra_SCZ>HC_ and Inter_SCZ>HC_), more discernable trends emerge. First, the general patterns of hyper-activation are exaggerated under conditions of inter-hemispheric communication (Fig. 3b and Table 4), where clusters with greater extent are observed. Second, we now observed significant hyper-activity in multiple mid-line sites in the corpus callosum, a functional discovery that perfectly dovetails with the original motivations of the paradigm itself [28, 29]. Because callosal hyper-activations are not optimally displayed in transverse slices, we constructed Fig. 4.

Figure 4 recapitulates data from Fig. 3a, b, with an emphasis on visualizing hyper-activation sites in the corpus callosum. Accordingly, profiles of hyper-activation in each condition are overlaid on four contiguous mid-line sagittal slices of a segmented white matter mask. As is evident, the extent of hyper-activation is amplified in the inter-hemispheric condition (Fig. 4b). This effect implies that the more generalized network demands evoked by inter-hemispheric transfer drive the hyper-engagement of callosal pathways that specifically connect inter-hemispheric frontal and motor regions. We suggest that this effect in part provides a functional signature that conforms to structural effects that include hypo-intensity of callosal white matter [37], greater callosal diffusivity [27, 52], and structural connectivity [53].

## Discussion

A principal goal of this work was to extend the “inefficiency hypothesis” of SCZ [54] by exploring (a) fMRI estimated activations in white matter tracts during (b) a rapid visuo-motor integration task. Inefficiency is generally understood to refer to an overuse of resources to accomplish an outcome to the same or lower degree of proficiency, as in a normative group and is a hallmark of cortical reorganization in SCZ [55]. Moreover, the concept is associated with effortful processing in the context of learning and memory [20, 23]; these domains demand the heavy involvement of cortical regions like the dorsolateral prefrontal cortex and the dorsal anterior cingulate that are associated with executive processing and cognitive control [56, 57]. However, in neurobiology, inefficiency as a concept generalizes to clinical conditions of autonomous function. These functions include respiration [58], immune responses [59] and energy metabolism [60]. Therefore, logically, one *can* expect that even “fast” pre-attentive domains such as visuo-motor integration can evoke inefficient processing in the SCZ brain. Indeed, while recent evidence from the domain of object recognition provides evidence of efficiency loss in regions like the primary visual cortex [61], to our knowledge, no signatures of functional inefficiency have been detected in white matter tracts which form the principal pathways for information transmission across the brain. Thus, the dual novelty of our work lies in the combination of paradigm and analytical focus on white matter activations.

### White matter activations with fMRI and their relevance for SCZ

fMRI-derived white matter activations have been subject to debate because the sparse vascularization in white matter that can limit the amplitude and spatial resolution of the hemodynamic response, and reduced energy consumption in white (compared to gray) matter is assumed to preclude reliable detection of BOLD [62]. However, as has been persuasively argued [63], these constraints may be less limiting than believed. For example, at conventional field strengths (3 T), inter-hemispheric tasks such as the Sperry Task evoke reliable white matter activations in regions of the corpus callosum, though the sensitivity of detected clusters varies as a function of the applied hemodynamic response function used to model activation [64]. While obviously not directly attributable to the direct energy demands of synaptic transmission that are thought to be primary drivers of gray matter based BOLD [65], white matter axons exert metabolic demand [66], most likely from increased interstitial potassium (K+), that is a consequence of increased neuronal activity. Moreover, mitochondria along the length of axons require oxygen to produce ATP for cellular processes such as the re-establishment of ionic gradients after repeated action potentials [67]. Thus, it is likely that the BOLD response in white matter is sensitive to metabolic processes, but that the physiological correlates of this metabolism are partially distinct from gray matter.

As a “proof of concept” demonstration, multiple independent studies have reported white matter activations under a variety of conditions, suggesting that characterization of white matter engagement might greatly enhance our understanding of brain connectomics [32, 68]. These results have been derived in a variety of experimental contexts, samples and EPI acquisition techniques (echo-planar or spin-echo imaging) [31, 69–75]. White matter activations are systematically modulated by experimental context which itself is compelling evidence for the functional reality of these fMRI-measured activations [13]. As with changes in the functional response properties in gray matter, white matter activations are responsive to changes in basic motor proficiency following training, an effect that has been attributed to microstructural increases in white matter tract myelination [76] that have been detected following motor training. Finally, white matter activations follow fiber tracts within structures including the corpus callosum, further evidence that they constitute a legitimate functional signature of brain function [77, 78].

Given that local fMRI activity is generally related to metabolically induced demand following neurophysiological engagement [65], white matter activity appears to reflect tract-specific responses to neural activity, and may reflect functional echoes of information transmission across the cortex. Accordingly, our observed hyper-engagement across conditions of *both* intra- and inter-hemispheric transfer (Fig. 3) strongly motivates the inference that basic visuo-motor integration in SCZ is inefficiently achieved. Furthermore, the observed behavioral effects (Fig. 2), confirm that behavioral processing in patients is slower, and therefore *less* efficient. There are substantial bases for constraining these interpretations from other recent studies as well. When correlation tensors are built to quantify correlational anisotropy in white matter fMRI signals, the directional anisotropy of these functional tensors broadly conforms to diffusion-based tensors [75]. Moreover, the pathways are strongly evoked under basic visual stimulation, and task-related BOLD-based fMRI changes occur synchronously with the temporal pattern of stimuli. It is therefore reasonable to impute, that our observed fMRI activations reflect functional signatures of the brief visual stimulation induced during the Poffenberger paradigm, as visual information passes through longitudinal white matter tracts to motor areas where responses are initiated [79, 80]. Notably, inter-hemispheric transfer (Fig. 3c), a condition that places greater demands on network integration [29] (and which was a key motivation for the development of the Poffenberger paradigm), evokes greater extent of white matter hyper-activation in SCZ. This effect is broadly consistent with evidence that inter- more than intra-hemispheric transfer evokes a greater extent of white matter in healthy right-handed participants during uni-manual responding [32].

Further confirmation for this inference emerges from the assessment of specific activation differences in the corpus callosum (Fig. 4). As *the* principle inter-hemispheric commissure, the corpus callosum contains both small and large diameter fibers [81]; the density of small diameter fibers is highest in the anterior-genu and posterior-most (splenium) sub-regions of the structure, sub-regions that bridge the frontal- and temporal cortices. In comparison, a higher frequency of large diameter fibers is found in the more central sub-regions, the body and the isthmus, and may facilitate the rapid transfer of inter-hemispheric information during the early phases of sensory processing [82]. Thus, callosal axons preferentially drive post-synaptic targets, precisely because they are coupled to specific inputs. This organization of inter-hemispheric fibers in the structure may endow the cortical network with a degree of condition-specific flexibility [83]. Accordingly, the spatial loci of inter-group differences in the corpus callosum in each of the conditions assume significance. As seen in Fig. 4, both the intra- and inter-hemispheric conditions evoked hyper-activation in the genu and the splenium in patients. These effects suggest that in patients, there is inefficient hyper-engagement of callosal structures more closely associated with small fibers [84] regardless of experimental condition. However, as specifically seen in Fig. 4b, hyper-active loci in the body and the isthmus are only evoked during inter-hemispheric transfer, suggesting a unique interaction between task characteristics, patterns of callosal engagement, and the disease process of SCZ.

There was a notable absence of hypo-activation in SCZ participants regardless of condition, an effect that is inconsistent with early accounts of hypo-functionality but clearly consistent with more recent meta-analyses. In a meta-analysis that included >300 studies, Crossley and colleagues found that hyper-activations in SCZ are widely distributed across the brain [85], and more saliently associated with brain network “hubs” that connect multiple brain regions. Inefficient engagement of central brain nodes reflects responses in a compromised functional system, that presumably is attempting to compensate for the relative failure in the engagement of other peripheral regions [86].

### Relationship to studies of white matter microstructure and metabolism in SCZ

Alterations in white matter micro- and macro-structure have been extensively associated with the pathophysiology of SCZ [2, 38, 87]. For example, tract-based and diffusion tensor imaging studies of the corpus callosum have associated alterations in the genu with impairments in neurocognition and aberrant social functioning [88, 89] as well as alterations in diffusivity and fractional anisotropy [90, 91]. More generally, a loss of white matter connectivity has been associated with core emergent features of psychosis, specifically because this loss impacts conscious access to perceptual states [92]. However, the relationship between structure and function in pathology is frequently obscure, because the disrupted function is frequently associated with intact structure and vice versa [93].

Accordingly, it is useful to refer to the existing literature on white matter metabolic mapping in SCZ. In some of the earliest such examples, Buchsbaum and colleagues investigated alterations in metabolic consumption in several white matter structures using FDG-PET, a gold standard measure of brain metabolism [94, 95]. Compared to controls, SCZ patients were characterized by significantly higher metabolic rates in the corpus callosum, frontal, superior longitudinal, and temporal lobe white matter circuit, findings that have been partially replicated in more recent studies [96]. Moreover, white matter metabolism is inversely associated with fractional anisotropy in SCZ, suggesting that a decrease in the structural integrity of white matter results in an increase in indices of brain engagement [97]. Thus, observed functional inefficiency is a logical expression of unsurmountable structure brain deficits. This conceptualization provides a very lucid and intuitive framework that links the many aspects of “dys-connection” in SCZ [10], such that observed dys-*function* emerges from a compromised structural substrate, but is contextually evoked by task or condition-related specificity [98, 99].

The novelty of our approach and results notwithstanding, we note some empirical limitations. For example, while patients performed more poorly than controls, post-hoc multivariate analyses did not reveal significant associations between clinical variables and response latencies in patients. While such associations would be predicted by prior studies of general cognitive functioning, memory, and ultimately basic visuo-motor skills [100–102], our sample size was optimized for fMRI and not neuropsychological analyses. In addition (and as previously alluded to), we do not have specific mechanistic explanations for the observed inter-group differences in white matter engagement. However, in our defense invoke the relatively contextual specificity of the results, particularly in the corpus callosum (Fig. 4), and note that our approach is consistent with the as yet small, but growing focus on the meaning of white matter activations detected with fMRI [103].

## Conclusion

SCZ appears to affect everything in the brain, yet it also emerges from everything that *is* affected in the brain [104]. This aspect was perfectly intuited by Kraepelin in his very early conceptualizations of the disease [105], and he anticipated subsequent discoveries in neuroscience that provide operational evidence for dys-connection. A leading question for researchers now, is not if SCZ is a dys-connection, but how it is so. We suggest that evidence of white matter hyper-engagement induced during basic visuo-motor processing constitutes novel evidence for the extent of and expression of this dys-connection syndrome. Future studies can help elucidate inter-relationships between white matter hyper-engagement, network connectomics, and the loss of white matter structural integrity in the illness. Multi-modal acquisitions and multiple analytical approaches may hold the key to deciphering a fuller picture of the pathophysiology of SCZ.
